# Attentional Measures of Memory in Typically Developing and Hypoxic–Ischemic Injured Infants

**DOI:** 10.3390/brainsci10110823

**Published:** 2020-11-06

**Authors:** Jennifer B. Wagner, Adeline Jabès, Agatha Norwood, Charles A. Nelson

**Affiliations:** 1 Department of Psychology, College of Staten Island, City University of New York, Staten Island, NY 10314, USA; 2 Department of Psychology, The Graduate Center, City University of New York, New York, NY 10016, USA; 3 Institute of Psychology, University of Lausanne, 1015 Lausanne, Switzerland; adeline.jabes@unil.ch; 4 Pediatric Medical Group of New Mexico, Presbyterian Hospital, Albuquerque, NM 87106, USA; agatha.norwood@gmail.com; 5 Division of Developmental Medicine, Boston Children’s Hospital, Harvard Medical School, Boston, MA 02215, USA; charles.nelson@childrens.harvard.edu; 6 Harvard Graduate School of Education, Harvard University, Cambridge, MA 02138, USA

**Keywords:** infancy, memory, eye-tracking, VPC, relational memory, hypoxic–ischemic injury

## Abstract

Hypoxic–ischemic injury (HII) at birth has been found to relate to differences in development, including decreased memory performance. The current study assessed recognition memory in 6- and 12-month-old HII infants and typically developing (TD) infants using two eye-tracking paradigms well suited to explore explicit memory processes early in life: visual paired comparison (VPC) and relational memory (RM). During the VPC, infants were familiarized to a face and then tested for their novelty preference immediately and after a two-minute delay. At 6 months, neither HII nor TD showed a VPC novelty preference at immediate delay, but at 12 months, both groups did; after the two-minute delay, no group showed a novelty preference. During RM, infants were presented with blocks containing a learning phase with three different scene–face pairs, and a test phase with one of the three scenes and all three faces appearing simultaneously. When there was no interference from other scene–face pairs between learning and test, 6-month-old TD showed evidence of an early novelty preference, but when there was interference, they revealed an early familiarity preference. For 12-month-old TD, some evidence for a novelty preference during RM was seen regardless of interference. Although HII and TD showed similar recognition memory on the VPC, when looking at RM, HII infants showed subtle differences in their attention to the familiar and novel faces as compared to their TD peers, suggesting that there might be subtle differences in the underlying memory processing mechanisms between HII and TD. More work is needed to understand how these attentional patterns might be predictive of later memory outcomes.

## 1. Introduction

Hypoxic–ischemic injury (HII), which occurs in 1–6/1000 live full-term births, results in increased risk for neonatal mortality and later neurodevelopmental disabilities [1,2]. The hippocampal formation, a well-known functional entity fundamental for explicit memory functions (e.g., [3,4,5]), is particularly susceptible to perinatal HII (for reviews, see [6,7]). Although many previous human studies have demonstrated atrophy of the hippocampal formation and memory impairments following HII [8,9,10,11,12,13,14,15,16], these alterations have not been noted to occur until school age [17]. One explanation for this could be that the hippocampal formation exhibits a late maturation lasting into young adulthood, with major morphological changes occurring within the first 4 years of age in humans [18], so it is not until this point that the memory differences might become evident. Conversely, memory impairments in children who have experienced perinatal HII may be present from the time of the injury but may go unnoticed until they enter school because relatively few demands are placed on memory during infancy or early childhood. Moreover, most studies have used standardized scales to assess episodic memory and might have missed other explicit memory functions emerging at younger ages [18].

Tracking of visual attention in nonverbal infants offers a reliable monitoring and quantitative evaluation of behavior, and this has been used to assess early-emerging memory functions, including both recognition memory and basic relational memory (for a review, see [18]). Recognition memory has been most often evaluated using the visual paired comparison (VPC) behavioral task. This task involves familiarizing the infant to a visual stimulus for a fixed period of time and subsequently testing the infant by showing the familiarized stimulus next to a novel stimulus at various retention intervals. Memory is inferred if the infant shows preferential looking, greater than is expected by chance, to one stimulus over the other, typically a preference toward the novel stimulus [19] (but see also [20]). Prior studies have used the VPC task to demonstrate visual recognition memory across time delays at various infant ages. With extensive familiarization, a preference for novel faces can be observed in 3- to 6-day-old neonates after a 2-min delay [21] and in 3- and 6-month-old infants after a 24-h delay [22]. Six-month-olds exhibited a preference for novel patterns after a 48-h delay, and for novel faces, after a 2-week delay [23]. Five- and 9-month-olds, but not 3-month-olds, familiarized for 120 s with a toy moving in one of two different orientations, exhibited a preference for the novel orientation after a 5-min delay, no preference after a 2-week delay, and a familiarity preference after a 1-month delay [24]. With a familiarization of only 10 s, 12-month-olds and 2- and 3-year-olds exhibited a preference for a novel cartoon-like face after no delay, but only 2- and 3-year-olds showed a novelty preference after 24-h and 1-week delays [25]. Through use of the VPC task, all of these studies demonstrated the presence of visual recognition memory in infants from a few days after birth depending on the procedure used, with an overall trend toward retention over progressively longer time delays with increasing age.

Eye-tracking techniques have added to the study of early infant memory by allowing for a higher level of temporal and spatial resolution when examining visual attention than traditional measures afford (for discussion, see [26]). Multiple studies have used eye-tracking to assess basic relational memory function in infants. Richmond and Nelson (2009) [27] adapted eye-tracking measures of relational memory that were designed for use with adult amnesiacs [28] to study relational memory during the first year of life. During a learning phase, they exposed 9-month-old infants to three arbitrarily paired scenes and faces (e.g., a face overlaid on a pond with a water lily). Then, during the test phase, one of the three scenic backgrounds was again presented, this time with all three faces superimposed. Each of the three faces were part of a scene–face pairing during learning and were therefore familiar, but only one was previously presented with that particular scene. Richmond and Nelson (2009) [27] showed that 9-month-old infants looked preferentially at the face that matched the test background for trials using both the last scene–face pair presented during learning (Lag 0, or no delay), and the first scene–face pair presented during learning (Lag 2, 20–30 sec delay), but these preferences were only seen at the start of the test phase. Chong, Richmond, Wong, Qiu, and Rifkin-Graboi (2015) [29] showed that, with similar procedures, 6-month-olds looked preferentially at the toy matching the test background, but the extent of this preference was dependent on attention during the task. Specifically, when using strict inclusion criteria for attention during test trials, 6-month-olds showed a preference for the matching toy on Lag 0 trials at the start of the test phase, but no preference during Lag 2 trials. When minimum looking time criteria were loosened, Chong et al. (2015) [29] found no evidence of looking preference during Lag 0 trials, but a sustained preference for the matching toy throughout the test phase for Lag 2 trials. Furthermore, consistent with the strict inclusion criteria findings by Chong et al. (2015) [29], Richmond and Power (2014) [26] also showed that 6-month-olds exhibited a preference for the face matching the scenic background for Lag 0 but not for Lag 2 trials. This work tested 12-month-olds as well and found that this older age group did not show a preference for the matching face on either Lag 0 or Lag 2 trials, though these infants did show a preference *away* from the matching face for Lag 2 trials within the first 250 ms. The authors suggested that the nature of these representations might change with age, becoming less “unitized” around the first year of life and impacting visual behavior. Accordingly, Koski, Olson, and Newcombe (2013) [30] showed that 4-year-olds’ preferential looking at the correct background–face pair was at chance level. 

Overall, measures of visual attention in infancy have provided a window into the very early developmental profile of some explicit memory functions, and in some cases these measures have been used to assess the impact of perinatal adverse experience on the hippocampal formation integrity. For example, it has been shown that infants of diabetic mothers, a group at risk for hippocampal damage [31], did not show any impairments in recognition memory during the first year of life despite electrophysiological evidence of subtle perturbations [32,33]. Similarly, our previously published work looked at electrophysiological and VPC indices of memory in 12-month-old infants who experienced HII and found that this group did not differ from typically developing infants on the VPC, exhibiting a novelty preference only in a no delay condition, but not in a 2-min or 24-h delay condition [34]. However, aspects of the electrophysiological signature during testing differed between groups, suggesting different neural mechanisms might underlie memory performance in HII. The current study extends our previous report to study two attentional tests of memory development in 6- and 12-month-old infants with a history of perinatal HII and typically developing infants: (a) visual recognition memory to a single familiarized stimulus at immediate and delayed testing through a VPC, and (b) relational memory of associations between two items as remembered over immediate and delayed testing. These memory tests examine two different aspects of memory, with the VPC as a more basic recognition task and the RM as a more complex associative learning task. Memory performance on these two tests was examined through patterns of visual attention recorded on an eye-tracking monitor. The aim of this study was to test early explicit memory functions in 6- and 12-month-old HII infants and to compare developmental profiles between typically developing infants and those with a history of HII. Based on findings from Norwood et al. (2014) [34], we hypothesized that the groups would not differ on the basic recognition memory task (VPC), but that the RM task, which captures a more elaborate form of memory processing, might show differential patterns of responding between the two groups. 

## 2. Method

### 2.1. Participants

The final sample consisted of 114 infants across two age groups (6 or 12 months), and this included 90 healthy, typically developing infants (TD; 6 months, *n* = 47, mean age = 197 days, *SD* = 12 days; 25 male; 12 months, *n* = 43, mean age = 383 days, *SD* = 16 days; 20 male) and 24 infants who experienced a hypoxic–ischemic injury in the perinatal period (HII; 6 months, *n* = 12, mean age = 205 days, *SD* = 16 days; 8 male; 12 months, *n* = 12, mean age = 382 days, *SD* = 14 days; 8 male). Inclusion criteria for all infants was birth at greater than or equal to 35 weeks of gestational age. Mean gestational age did not differ between the two groups (HII: *M* = 3.4 days early, *SD* = 8.7; TD: *M* = 2.5 days early, *SD* = 9.6; *p* = 0.68), but weight at birth was significantly lower for HII (*M* = 7.0 lbs, *SD* = 0.6) as compared to TD infants (*M* = 7.7 lbs, *SD* = 1.1; *t*(106) = 3.12, *p* = 0.002). Preliminary analyses examined HII infants alongside a subset of TD infants matched on gestational age and weight, and findings remained largely unchanged. With this, the reported results compared HII infants to the full sample of TD infants in order to increase power in the current analyses. 

HII infants were recruited from the neonatal neurology clinic at Boston Children’s Hospital. To qualify for the present study, HII infants had to have exhibited clinical signs and/or symptoms of perinatal hypoxic injury, which included seizures or two of the following symptoms lasting for more than 24 h: abnormal consciousness, difficulty maintaining respiration, abnormal tone/reflexes, or feeding difficulty of presumed central origin. The HII infants included in the current study suffered mild–moderate severity of illness, with all but one infant in Sarnat stage range I-II. Additional information available on severity of illness for the HII 6-month and 12-month groups is detailed in Table 1, including number of subjects who required therapeutic hypothermia and/or suffered seizures, 1 min and 5 min Apgar scores, and initial blood pH. Appendix A details the clinical characterization for each HII participant. Exclusion criteria were any chronic fetal/infant factors such as IUGR, maternal drug use, maternal diabetes, metabolic disorder, congenital malformations, or severe quadriplegia or significant abnormality in vision/eye movements. TD participants were recruited from the Research Participant Registry of the Laboratories of Cognitive Neuroscience at Boston Children’s Hospital.

HII and TD participants were included in the final sample if they had sufficient data from either of the two eye-tracking tasks. Nine infants (five 6-month TD, one 12-month TD, and three 12-month HII) were excluded because they did not meet inclusion criteria for either eye-tracking analysis (criteria detailed under Data Processing). An additional 6-month HII infant was not included due to errors in data export. Finally, two HII 12-month-old infants were excluded from subsequent analyses due to severe motor and visual impairment. 

Project approval was obtained from the Institutional Review Board of Boston Children’s Hospital and informed consent was obtained by the parents of each infant participant. The included sample was predominantly White (78%) and Non-Hispanic (84%), with 70% of families reporting household incomes above $70,000, and 89% of highest paternal education and 96% of highest maternal education reported as 4-year college degree or higher. Full demographic information for the two groups at each age can be found in Appendix B. 

### 2.2. Stimuli

Stimuli for the VPC task consisted of color photographs of female faces displaying neutral expressions. Each woman was seated in front of a gray background and wearing a gray cloth to cover their clothing. Face images were taken from a database of women who participated in other studies with their infants and signed a release for use of their image in future research. All of the faces used were Caucasian females with neutral expressions and none contained glasses or other accessories. Size and luminance of all stimuli were also matched to ensure that high- and low-level stimulus differences could not influence the present study (for sample stimuli, see [34]). For the RM task, each block contained three scenes and three faces. The face stimuli were taken from the same database of neutral female faces as used in the VPC, but no faces overlapped between the two tasks. The scene images in the current study were from stimuli used in previous RM tasks (for sample stimuli, see [27]). 

### 2.3. Apparatus 

Participants were seated on a chair in front of a 17″ TFT Tobii T60 monitor (Tobii Technology AB; www.tobii.com). Images were presented on the monitor using Tobii Studio software. During stimulus presentation, the Tobii monitor recorded gaze location for both eyes based on the reflection of near-infrared light from the cornea and pupil. Gaze information was sampled at a frequency of 60 Hz. Manufacturer specifications for the monitor included an accuracy of 0.5 degrees of the visual angle and a tolerance of head movements within a range of 44 × 22 × 30 cm (though see work by [35] showing accuracy closer to 1–1.5 degrees for infants). 

### 2.4. Procedure 

For 6- and 12-month families who were part of a larger testing protocol (see [34]), infants were presented with both the VPC and RM tasks during their visit. For a subset of 6-month TD that were not enrolled in the larger testing protocol due to time constraints (*n* = 18), the RM task was administered at the start of a separate testing session and no VPC was administered. In all cases, the infants were seated on their caregiver’s lap on a chair in front of a Tobii T60 monitor in a sound-shielded testing room with dim lighting. The chair was positioned such that each participant’s eyes were approximately 60 cm from the monitor. Before beginning the eye-tracking tasks, participants completed a calibration procedure to ensure the eye-tracker was adequately tracking gaze. In this calibration procedure, a red dot appeared at five locations: each of the four corners of the monitor and the center of the screen. Following calibration, the Tobii Studio program reported whether the eye-tracker successfully picked up gaze at the five locations. If calibration was successful, the experimental procedure was begun. If calibration was unsuccessful, the monitor and chair were adjusted and the calibration procedure was re-run until it successfully picked up on all five locations of gaze. 

Following calibration, infants were first presented with the VPC task (except the RM-only 6-month TD subset). During all phases of the VPC, faces were presented side-by-side. During the VPC familiarization phase, infants were presented with the same unfamiliar female face on both the left and right side of the screen for 25 s. Infants were then tested using the VPC at two delays during the session: no delay, which immediately followed familiarization, and two-minute delay, which was two minutes after familiarization. For each of the VPC tests, infants were shown the familiar face next to a novel face for a total of 20 s, with the left/right position of the faces switching sides after 10 s. At each VPC comparison delay, infants saw a unique face paired with the familiarization face.

After completing the VPC task, infants were presented with the RM task based on the study design of Richmond and Nelson (2009) [27]. For the 6-month TD who did RM only, this task was the first task after calibration, as the VPC was not included. For each block within the RM task, infants saw three learning trials followed by a test trial. The learning trials consisted first of a scene presented for 3000 ms, then the scene remained in the background with a face appearing centrally for 5000 ms. The test trials then consisted of either the 1st scene or the 3rd scene from the learning phase presented again for 3000 ms, followed by all three faces from the learning trials simultaneously for 5000 ms, arranged in three novel positions: left, right, and bottom (see [27]). Test trials showing the 1st scene, referred to as Lag 2 trials, tested memory following two interfering scene–face pairs, while test trials showing the 3rd scene, referred to as Lag 0 trials, tested memory after no interfering scene–face pairs. Positioning of the match face was counterbalanced across trials. Stimuli were triggered by the experimenter when infants were attending to the screen, and infants were presented with RM blocks until they saw 12 trials (six Lag 0, six Lag 2), or until they became fussy or inattentive. 

### 2.5. Data Processing

Before the eye-tracking data were exported from Tobii Studio, areas of interest (AOIs) were drawn on the stimuli, enabling the subsequent analysis of gaze data within these particular regions. After export, data files were run through a custom-made Python script (Python programming language; www.python.org) that extracted and summed gaze duration for each AOI on each trial for each eye-tracking task. 

For the VPC, AOIs were created for each picture that encompassed the face and gray background and were labeled as either familiar face or novel face. Each participant’s eye-tracking data were exported from Tobii Studio, with time samples identified in which gaze fell within one of the face AOIs. Using gaze duration to the familiar face during familiarization, two variables were calculated for each infant to determine if they had sufficient and unbiased looking during this initial phase: (a) total time on familiar face (summing time on the familiarized face that appeared on both the left and right sides of screen), and (b) side bias, calculated as total time on the familiarized face on the left side divided by total time on the familiarized face on the left plus the right sides of the screen. Infants were included in subsequent VPC analyses if (a) during familiarization, they looked at the face more than 20% of the time (i.e., 5 s out of the 25 s length of familiarization) and had a side bias no greater than 85% to either side, and (b) during one or both test delays (no delay, two minutes), they looked to the faces more than 20% of the time (i.e., 4 s out of the 20 s full trial). This inclusion criteria took a less conservative approach than that of our past work reporting on a subset of the current 12-month sample (i.e., 30% looking, see [34]) in order to maximize included infants across ages. The current criteria resulted in no delay data from 22 6-month TD, 10 6-month HII, 36 12-month TD, and 10 12-month HII, and two-minute delay data from 19 6-month TD, 10 6-month HII, 34 12-month TD, and 10 12-month HII.

The VPC data were analyzed for novelty preference by calculating the proportion of time on the novel face divided by the total time on the novel and familiar faces combined and multiplying by 100. This novelty percentage was calculated across the full 20 s of a given delay for the no delay condition and for the two-minute delay condition. 

For the RM task, AOIs were drawn around the scene image as well as the face images. For a block to be included in analysis, infants had to look at each scene–face pair during the learning trials, with criteria set for looking >500 ms on the scene when initially presented (out of 3000 ms), and >500 ms on the face presented in the center of the scene (out of 5000 ms). Additionally, on the test trial for that block, infants had to look for >500 ms at the scene when initially presented (out of 3000 ms) and >500 ms summed across the three faces appearing with the scene (out of 5000 ms). Participants had to contribute at least two trials for Lag 0 or Lag 2 to be included in statistical analyses for the RM task. The criteria used for included infants in the current study was modeled after the more inclusive criteria detailed in Chong et al. (2015) [29], ensuring a balance between sufficient encoding during learning and attention during the test trial, while still maximizing the number of included infants. This criteria resulted in Lag 0 data from 31 6-month TD, six 6-month HII, 31 12-month TD, and 10 12-month HII, and Lag 2 data from 30 6-month TD, 11 6-month HII, 32 12-month TD, and 11 12-month HII.

On average, infants contributed 3.6 Lag 0 trials (*SD* = 1.1) and 3.4 Lag 2 trials (*SD* = 1.3). For each block’s test trial, proportion of time on the matching face out of the total time on all three faces was calculated first across the full 5000 ms time bin (full bin: 0–5000 ms). Based on past findings by Richmond and colleagues of initial preferences that change across the trial duration [26,27,29], this proportion was also examined in 1000 ms time bins (bin 1: 0–1000 ms, bin 2: 1000–2000 ms, bin 3: 2000–3000 ms, bin 4: 3000–4000 ms, bin 5: 4000–5000 ms) and using 500 ms time bins within the first 1000 ms (bin 1.1: 0–500 ms, bin 1.2: 500–1000ms). For each participant, the average proportion of time on the matching face was calculated for Lag 0 and Lag 2 test blocks. 

### 2.6. Statistical Analysis

For VPC analyses, 2 (Age: 6 months, 12 months) × 2 (Group: TD, HII) ANOVAs were conducted for the no delay and the two-minute delay conditions. A series of one-sample *t*-tests was then conducted to look for novelty preferences above chance (50%) for each delay condition for TD and HII infants at each age. 

For RM analyses, 2 (Age: 6 months, 12 months) × 2 (Group: TD, HII) ANOVAs were conducted for Lag 0 and Lag 2 trials across the full time window (0–5000 ms). Next, a series of one-sample *t*-tests were used to look for attentional patterns to the matching face that differed from chance (33%) for Lag 0 and Lag 2 trials for TD and HII at each age. These *t*-tests were conducted first for the full time window (0–5000 ms), then for the five 1000 ms time bins (0–1000 ms, 1000–2000 ms, 2000–3000 ms, 3000–4000 ms, 4000–5000 ms), and for the two initial 500 ms time bins (0–500 ms, 500–1000 ms). Independent sample *t*-tests were then used to examine differences in attention to the matching face as a function of TD vs. HII at each age for each 1000-ms and 500-ms time bin at Lag 0 and Lag 2.

Although a subset of participants contributed data at both 6 and 12 months, the present study focuses on cross-sectional analyses that treat age as a between-subjects factor.

## 3. Results

### 3.1. Visual Paired Comparison

There was a main effect of age for novelty preference in the no delay condition (*F*(1,74) = 7.313, *p* = 0.008, η_p_^2^ = 0.09), with a stronger novelty preference at 12 months (*M* = 58.9%, *SD* = 11.6%) as compared to 6 months (*M* = 49.4%, *SD* = 17.1%). There was no interaction between age and group, but a marginal effect of group was seen (*F*(1,74) = 2.917, *p* = 0.092, η_p_^2^ = 0.038), with a trend suggesting greater novelty preference in HII (*M* = 58.7%, *SD* = 11.9%) as compared to TD (*M* = 53.6%, *SD* = 15.5%). Both groups at 12 months showed a novelty preference greater than chance (12-month TD: *t*(35) = 3.97, *p* < 0.001, *d* = 0.66; 12-month HII: *t*(9) = 3.55, *p* = 0.006, *d* = 1.12), but 6-month-old HII and TD did not show behavior different from chance (*p*s ≥ 0.25). 

There were no significant main effects or interaction for novelty preference in the two-minute delay condition (*F*s < 0.85, *p*s > 0.36). Looking did not differ significantly from chance for TD or HII at either age (*p*s > 0.48). Table 2 shows novelty percentage for each delay at each age separated by TD and HII. 

### 3.2. Relational Memory

#### 3.2.1. Lag 0 Trials

There were no main effects or interaction for Lag 0 trials across the full time window (0–5000 ms) (*F*s < 0.78, *p*s > 0.38). None of the four groups of infants showed looking to the matching face that differed from chance for the full time window (*t*s < 1.3, *p*s > 0.20; see Table 3). 

**Figure 1:**
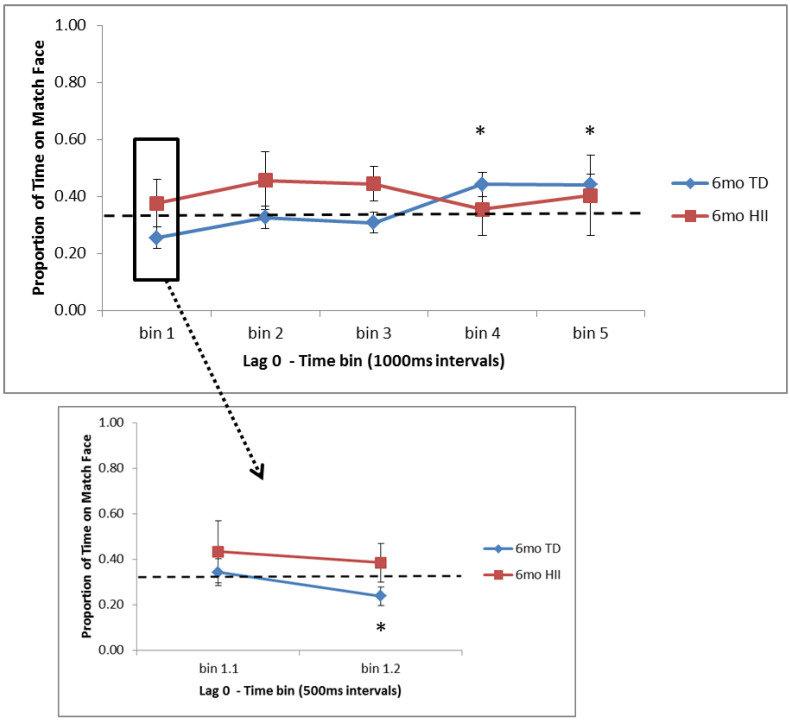
Proportion of time fixating the matching face for Lag 0 trials for the five 1000 ms time bins (bins 1–5) and the two 500 ms bins in the first 1000 ms (bins 1.1 and 1.2) for 6-month-old typically developing infants (TD) and hypoxic–ischemic injured infants (HII). Looking to the match face was significantly higher than expected by chance for 6-month TD infants in bins 4 (3000–4000 ms) and 5 (4000–5000 ms), and significantly lower than expected by chance in bin 1.2 (500–1000 ms). Chance is illustrated with a dashed line; error bars indicate the standard error of the mean. * *p* < 0.05

**Figure 2:**
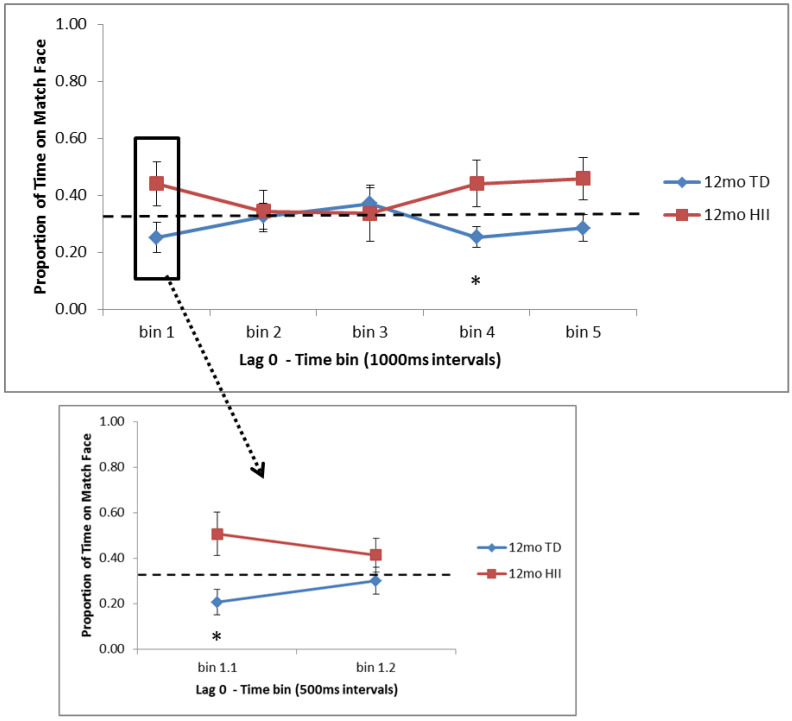
Proportion of time fixating the matching face for Lag 0 trials for the five 1000 ms time bins (bins 1–5) and the two 500 ms bins in the first 1000 ms (bins 1.1 and 1.2) for 12-month-old typically developing infants (TD) and hypoxic–ischemic injured infants (HII). Looking to the match face was significantly lower than expected by chance for 12-month TD infants in bin 4 (3000–4000 ms) as well as in bin 1.1 (0–500 ms). Chance is illustrated with a dashed line; error bars indicate the standard error of the mean. * *p* < 0.05.

Time bin analyses revealed that 6-month TD spent significantly more time on the matching face than expected by chance in the last two bins (3000–4000 ms, *p* = 0.013, *d* = 0.48; 4000–5000 ms, *p* = 0.004, *d* = 0.56). Within the first 1000 ms, however, 6-month TD showed significantly less time on the matching face than expected by chance for the 500–1000 ms bin (*p* = 0.031, *d* = 0.41), reflecting a novelty preference near the start of the trial that shifts to a familiarity preference by the end (see Figure 1). For 12-month TD, no preference for the matching face was found, and instead, a preference for novelty was seen near the start of the trial (0–500 ms, *p* = 0.038, *d* = 0.40) and in the fourth time bin (3000–4000 ms, *p* = 0.05, *d* = 0.37; see Figure 2). At 12 months, HII showed the opposite pattern for 0–500 ms, with a trend towards greater time on the match face during this initial bin (*p* = 0.10, *d* = 0.58). No other one-sample *t*-tests were significant.

Differences between TD and HII for Lag 0 trials were examined using independent-sample *t*-tests separately for 6 and 12 months. No differences were found for comparisons between 6-month TD and 6-month HII (Figure 1). At 12 months, significant differences were found between TD and HII for the initial 0–500 ms bin (*p* = 0.011, *d* = 0.97) as well as for bin 4 (3000–4000 ms, *p* = 0.024, *d* = 0.86). Additionally, marginal differences in attention to the matching face were seen between TD and HII 12-month-olds for bin 1 (0–1000 ms, *p* = 0.074, *d* = 0.67) and bin 5 (4000–5000 ms, *p* = 0.071, *d* = 0.68; Figure 2).

#### 3.2.2. Lag 2 Trials

**Figure 3:**
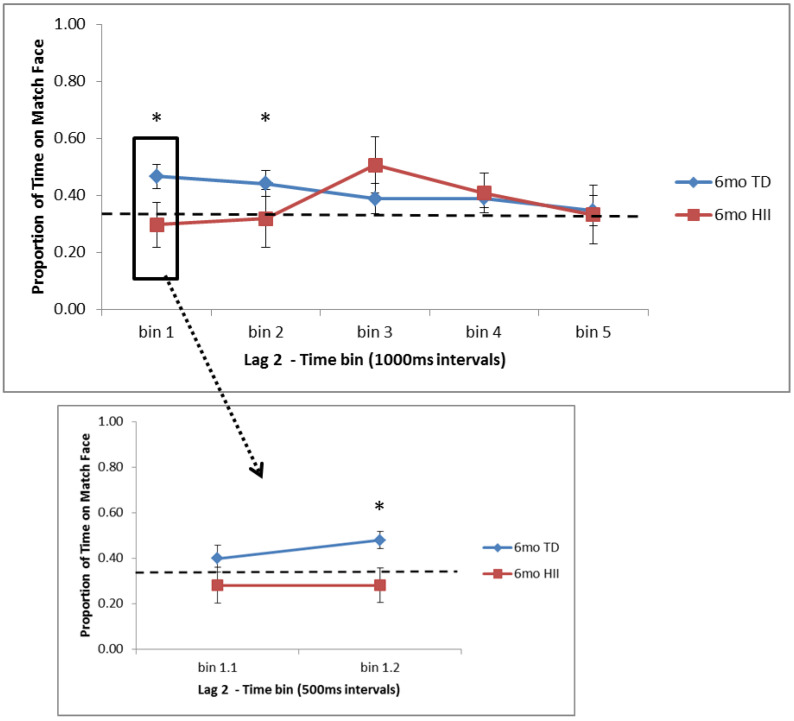
Proportion of time fixating the matching face for Lag 2 trials for the five 1000 ms time bins (bins 1–5) and the two 500 ms bins in the first 1000 ms (bins 1.1 and 1.2) for 6-month-old typically developing infants (TD) and hypoxic–ischemic injured infants (HII). Looking to the match face was significantly higher than expected by chance for 6-month TD infants in bins 1 (0–1000 ms) and 2 (1000–2000 ms) as well as in bin 1.2 (500–1000 ms). Chance is illustrated with a dashed line; error bars indicate the standard error of the mean. * *p* < 0.05.

**Figure 4:**
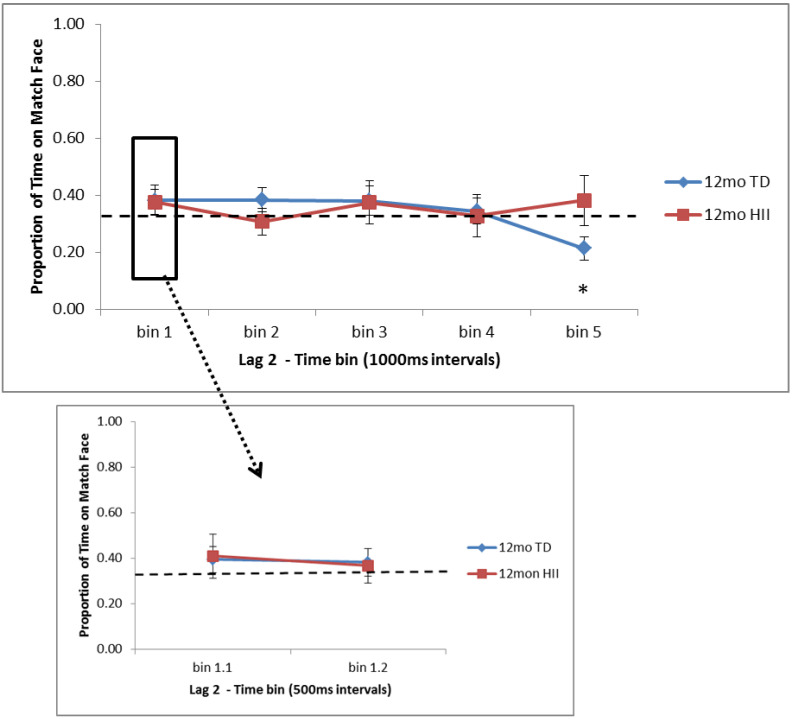
Proportion of time fixating the matching face for Lag 2 trials for the five 1000 ms time bins (bins 1–5) and the two 500 ms bins in the first 1000 ms (bins 1.1 and 1.2) for 12-month-old typically developing infants (TD) and hypoxic–ischemic injured infants (HII). Looking to the match face was significantly lower than expected by chance for 12-month TD infants in bin 5 (4000–5000 ms). Chance is illustrated with a dashed line; error bars indicate the standard error of the mean. * *p* < 0.05.

There were no main effects or interaction for Lag 2 trials across the full 5000 ms time window (*F*s < 2.4, *p*s > 0.13). For this full window, 6-month TD showed significantly more time on the matching face than expected by chance (*p* = 0.014, *d* = 0.48), but no other group showed a significant overall preference (see Table 3). Time bin analyses revealed that for 6-month TD, significantly more time than expected by chance was spent on the matching face in the first two 1000 ms bins (0–1000 ms, *p* = 0.003, *d* = 0.59; 1000–2000 ms, *p* = 0.017, *d* = 0.47), and this was also significant between 500–1000 ms (*p* = 0.001, *d* = 0.71; see Figure 3). A trend towards more time on the match face was seen for 6-month HII in the third time bin (2000–3000 ms, *p* = 0.104, *d* = 0.54). For 12-month TD, significantly less time was spent on the matching face in the final time bin (4000–5000 ms, *p* = 0.008, *d* = 0.50), reflecting a novelty preference, but no other time bin for 12-month TD or HII showed attention significantly different from chance (see Figure 4).

Differences between TD and HII for Lag 2 trials were examined using independent-sample *t*-tests separately for 6 and 12 months. At 6 months, significant differences were found between TD and HII for the initial 0–1000 ms bin (*p* = 0.05, *d* = 0.71) and, specifically, within the 500–1000 ms bin (*p* = 0.016, *d* = 0.89; Figure 3). No significant differences were found between attention to the matching face in 12-month TD and 12-month HII, though there was a marginal difference between groups for the final time bin (4000–5000 ms, *p* = 0.09, *d* = 0.63; Figure 4).

## 4. Discussion

The current study used eye-tracking to examine two types of explicit memory functions in 6- and 12-month-old infants who had experienced perinatal hypoxic–ischemic injury and a group of typically developing infants. The first task utilized a VPC task, testing infant recognition memory immediately after familiarization and after a two-minute delay. The second task examined RM, asking how infants bind arbitrary pieces of information (scene–face pairs) and how this is affected by different amounts of interference from learning until test, with memory tested for Lag 0 trials (no interference between learning and test) and Lag 2 trials (two sets of interfering stimuli between learning and test). Results showed no differences between HII and TD for VPC, but the RM task suggested preliminary patterns of attention to novelty and familiarity that might differentiate HII and TD in certain time windows. 

The VPC has been used across a large number of developmental studies to examine recognition memory across contexts and delays in infants and young children (see [20]). The current study found that (a) 6-month-old HII and TD showed no preference at an immediate or 2-min delay, (b) 12-month-old infants in both groups showed a novelty preference immediately after familiarization, but no preference after a 2-min delay, and (c) no significant group differences were found between HII and TD. A subset of 12-month-old HII and TD infants from the current sample were included in VPC analyses in Norwood et al. (2014) [34], where a novelty preference was also found only at immediate test for both groups. VPC studies have found wide variation in infant visual preferences, often pointing to novelty preferences soon after familiarization, null effects at intermediate time delays, and familiarity effects at extended delays (e.g., [36]; for discussion, see [37]). While the current study did find novelty preferences in 12-month-olds immediately after familiarization, no preferences were found for 6-month-olds on immediate test, nor for either age group following a 2-min delay, despite past work showing such preferences (see [20]). 

Future work is needed to clarify the variations in VPC testing conditions and analysis parameters that can lead to differential findings of infant novelty preferences. Studies have found that differences in familiarization time can lead to differences in attentional preferences (e.g., [38,39]), and in line with this, a post hoc look at the use of stricter familiarization requirements for the current sample (e.g., 40% or 50%) revealed that novelty preferences at both delay conditions will increase with this change in criteria. Another parameter to consider in understanding VPC attentional preferences in infants is how the time window analyzed might also lead to null effects (see [27] for discussion of this in RM studies). For example, an infant who detects the novel face at the start of a VPC test might look at that novel face initially, but if the test trial extends past a certain point, their subsequent attention to the familiar and novel faces might cancel out any initial preference seen. In the current study, each VPC test was 20 s in duration, with the positions of the novel and familiar faces switched after 10 s. A post hoc look at the use of the first 10 s of VPC rather than the full 20 s time window also showed that increasing novelty preferences would result. Systematic work in this area in the future will allow for a better understanding of how these VPC parameters influence infant attentional preferences. Nevertheless, our VPC data showed that infant attentional preferences did not differ between HII and TD infants, suggesting no disruption of early visual recognition memory function after HII at birth. 

For the relational memory task, the current study found that for typically developing infants, (a) 6-month and 12-month TD have an initial preference for novelty in Lag 0 trials (trials with no interference between learning and test), (b) after the initial novelty preference in Lag 0 trials, 12-month TD continue to show a novelty preference later in the trial as well, but 6-month TD shift to a preference for the matching face at the end of the trial, and (c) on Lag 2 trials (with two scene–face pairs interfering between learning and test), 6-month TD show an early preference for the matching face, while 12-month TD again show evidence of a novelty preference late in the trial. Across three studies, Richmond and colleagues [26,27,29] found evidence of memory for scene–face pairings in 6, 9, and 12-month-olds, though the patterns of attention differed based on age, Lag (0 vs. 2), and looking criteria for inclusion. Overall, these past studies suggest that 6- and 9-month-olds typically look longer to the matching face on test, and that this is most pronounced near the start of the test trial. They also suggest that 12-month-olds show less consistent preferences on this task, with only an initial novelty preference seen for the most challenging test condition (Lag 2; [26]). The current findings expand this work in typically developing infants, showing evidence for relational memory in both 6- and 12-month-olds when more inclusive looking criteria are used as compared to initial infant RM studies (see also [29]). For Lag 0 trials, both 6-month and 12-month TD show a very early preference away from the matching face, or a novelty preference, but for 6-month TD, this shifts back to a familiarity preference, and during Lag 2 trials, 6-month TD show evidence for a familiarity preference only. With 12-month TD showing novelty preferences in RM both here and in past work, further research will be needed to understand the parameters that might lead to differences in attentional patterns, including novelty, null, and familiarity preferences, across memory tasks with young infants, parallel to what was discussed with regard to the VPC.

As far as similarities and differences between HII and TD on RM, findings suggest that (a) 6-month HII do not differ from 6-month TD on attentional preferences for Lag 0 trials, but for Lag 2, 6-month TD show significantly more time on the matching face than HII at the start of the test trial, and (b) 12-month HII do not differ from 12-month TD for Lag 2 trials, but for Lag 0, 12-month TD show significantly less time on the matching face than 12-month HII at both early and later points in the test window. These findings suggest that although HII and TD show similar recognition memory as measured by the VPC, the RM task might provide a more nuanced picture of memory development in HII infants, indicating possible group differences in memory processing for HII infants in the first year of life. With this, the current RM task is in line with electrophysiological findings from Norwood et al. (2014) [34], showing subtle differences in patterns of responding between HII and TD infants. For the 6-month HII, the Lag 2 data suggest a preference towards the matching face that appears 2000 ms after the same preference appears in 6-month TD infants, a difference that will need to be explored further, but potentially might suggest that HII show a delay in processing the matching face. For the 12-month HII, Lag 0 trials show trends towards a preference for the matching face early and late in the trial, in contrast to 12-month TD, who show a novelty preference instead. This contrast between a preference for novelty at 12 months in TD and a trend towards a preference for familiarity in HII might suggest variation in the mechanisms underlying encoding during the learning phase that might lead to differential outcomes at test. For example, past work has posited that limited vs. extended exposure to a stimulus could influence patterns of novelty and familiarity preference in infancy (for discussion, see [20]). While preliminary, the current 12-month differences between HII and TD suggest that despite similar exposure during the learning trials, HII infants might need more processing time during learning to fully encode the scene–face pair and show a subsequent novelty preference at test, as the 12-month TD group did.

A number of limitations of the current study should be acknowledged. First, a series of *t*-tests was conducted in order to examine looking behavior against chance as well as between groups, but it was deemed too conservative to correct for multiple comparisons in the present analyses due to small sample sizes and the increased possibility of making a type II error (e.g., for discussion, see [40,41,42]). Future work with larger samples will be needed to extend the developmental and group-level differences found in the present study. Second, although findings suggest similarities between groups on the VPC faces task and differences on the RM scene–face task, because there was no separate VPC scene task, it cannot be ruled out that group differences on RM might be due to difficulty processing scenes as compared to faces. While previous studies have not uncovered visual processing differences for scenes as compared to faces in populations who experienced HII, further study is needed to rule out this possibility for the differences in infancy. Finally, the current analyses focused only on familiarity/novelty preferences based on overall looking time, in line with past studies using similar tasks. Future work would benefit from the use of additional eye-tracking metrics, such as patterns of saccades and pupillometry, as these measures could further highlight similarities and differences in memory processing between HII and TD infants. An additional area for further exploration relates to a potential trend illustrated in 6-month-olds during Lag 0 trials on RM: a potential shift from an initial novelty preference to a later familiarity preference within trials. The current sample was too small to sufficiently examine questions of these cross-over preferences, but it will be important for future research to address how these preferences shift, in a narrow sense, within a given type of trial, and in a broad sense, across different age groups. 

In summary, while past studies with adolescents and adults who experienced early HII have found impairments in various aspects of memory and attention [14,43], the current work is among the first to look for memory-related differences in the first year of life that could result from HII. Consistent with Norwood et al. (2014) [34], HII infants showed similar recognition memory on the VPC, but when looking at RM, HII infants showed subtle differences in familiarity and novelty preferences as compared to their typically developing peers. Future work is needed to understand how early similarities and differences in memory processing strategies following HII could be predictive of developmental outcomes. This work highlights the benefit of using eye-tracking measures to detect subtle patterns of memory processing in the first year of life, an approach that allows for a richer look at both typical development and potential differences in development based on early adverse experiences.

## Figures and Tables

**Table 1:** HII severity of illness.

	HII	
	6 Month (*n* = 12)	12 Month (*n* = 12)
Therapeutic hypothermia (*n*)	5	6
Sarnat stage		
Stage I (*n*)	5	5
Stage II (*n*)	6	5
Stage III (*n*)	0	1
Seizures (*n*)	6	5
1-min Apgar score (range)	1–5	0–5
5-min Apgar score (range)	0–8	1–8
Initial pH (range)	6.8–7.3	6.6–7.3

Note: HII: Hypoxic–ischemic injured infants; clinical records were unavailable for one 6-month HII and one 12-month HII, so only seizure data are included (from parent report).

**Table 2:** Percentage of time on novel face in TD and HII at 6 and 12 months for visual paired comparison.

	6-Month TD	6-Month HII	12-Month TD	12-Month HII
No Delay	*n =* 22	*n* = 10	*n* = 36	*n* = 10
Mean (SD)	47.4% (19.4)	53.8% (9.9)	57.4% (11.2)	63.6% (12.1)
*p*-value	0.54	0.26	<0.001 *	0.006 *
Two-minute Delay	*n* = 19	*n* = 10	*n* = 34	*n* = 10
Mean (SD)	49.0% (12.0)	52.9% (12.4)	50.6% (13.2)	53.3% (18.9)
*p*-value	0.71	0.48	0.78	0.59

Note: TD: Typically developing infants; HII: Hypoxic–ischemic injured infants; *p*-values are results from one-sample *t*-test comparing each value to chance (50%); * denotes significance of *p* < 0.05.

**Table 3:** Percentage of time on match face in TD and HII at 6 and 12 months during the full time window (0–5000 ms) for relational memory.

	6-Month TD	6-Month HII	12-Month TD	12-Month HII
Lag 0	*n =* 31	*n* = 6	*n* = 31	*n* = 10
Mean (*SD*)	36.0% (12.9)	37.0% (7.5)	30.7% (18.3)	34.8% (9.6)
*p*-value	0.21	0.25	0.49	0.58
Lag 2	*n* = 30	*n* = 11	*n* = 32	*n* = 10
Mean (*SD*)	40.7% (16.1)	37.3% (15.9)	32.3% (17.8)	33.4% (10.6)
*p*-value	0.014 *	0.39	0.83	0.93

Note: TD: Typically developing infants; HII: Hypoxic–ischemic injured infants; *p*-values are results from one-sample *t*-test comparing each value to chance (33%); * denotes significance of *p* < 0.05.

## Appendix A

**Table A1:** HII Infant Clinical Characterization.

Participant	TherapeuticHypothermia	Sarnat Stage	Seizures	1-min Apgar	5-min Apgar	Initial pH	Age (months) with Useable Data
1	no	II	yes				6
2	yes	III	no	2	2	7	12
3	no	I	no	1	6	6.96	6, 12
4	yes	II	yes	1	1	6.8	6, 12
5			yes				6, 12
6	yes	II	no	1	5	7.19	6
7	no	I	no	2	1	7.2	6
8	yes	II	yes	3	4	7.01	6, 12
9	no	II	yes	5	8		6, 12
10	no	I	no	1	4	6.8	6, 12
11	yes	II	yes	0	2	6.6	12
12	yes	II	yes	1	0	7.2	6
13	yes	II	no	4	7	7.07	12
14	no	I	no	2	7	7.25	12
15	yes	I	no	1	3	7.3	6, 12
16	no	I	no	4	7	7.15	6, 12

Note: HII: Hypoxic-ischemic injured infants; Clinical records were unavailable for one infant (Subject 5), so only seizure data is included (from parent report). For data to be useable at a given age point, sufficient attention was required for one or both eye-tracking tasks.

## Appendix B

**Table A2:** Sample demographics.

	6-Month TD	6-Month HII	12-Month TD	12-Month HII
Infant Ethnicity				
Hispanic	4.3% (*n* = 2)	16.7% (*n* = 2)	2.3% (*n* = 1)	8.3% (*n* = 1)
Non-Hispanic	80.9% (*n* = 38)	75.0% (*n* = 9)	90.7% (*n* = 39)	83.3% (*n* = 10)
(Not Reported)	14.9% (*n* = 7)	8.3% (*n* = 1)	7.0% (*n* = 3)	8.3% (*n* = 1)
Infant Race				
White	66.0% (*n* = 31)	91.7% (*n* = 11)	81.4% (*n* = 35)	100.0% (*n* = 12)
Asian	8.5% (*n* = 4)	8.3% (*n* = 1)	2.3% (*n* = 1)	
Black or African American	2.1% (*n* = 1)			
More Than One Race or Other	10.6% (*n* = 5)		16.3% (*n* = 7)	
(Not Reported)	12.8% (*n* = 6)			
Highest Paternal Education				
High School Graduate		8.3% (*n* = 1)	7.0% (*n* = 3)	8.3% (*n* = 1)
Some College/2-Year Degree	4.3% (*n* = 2)	33.3% (*n* = 4)	4.7% (*n* = 2)	33.3% (*n* = 4)
4-Year College Degree	23.4% (*n* = 11)	41.7% (*n* = 5)	20.9% (*n* = 9)	41.7% (*n* = 5)
Graduate or Professional Degree	57.4% (*n* = 27)	16.7% (*n* = 2)	65.1% (*n* = 28)	16.7% (*n* = 2)
(Not Reported)	14.9% (*n* = 7)		2.3% (*n* = 1)	
Highest Maternal Education				
High School Graduate		8.3% (*n* = 1)		8.3% (*n* = 1)
Some College/2-Year Degree	4.3% (*n* = 2)	16.7% (*n* = 2)	9.3% (*n* = 4)	16.7% (*n* = 2)
4-Year College Degree	23.4% (*n* = 11)	33.3% (*n* = 4)	18.6% (*n* = 8)	41.7% (*n* = 5)
Graduate or Professional Degree	59.6% (*n* = 28)	41.7% (*n* = 5)	72.1% (*n* = 31)	33.3% (*n* = 4)
(Not Reported)	12.8% (*n* = 6)			
Household Income				
Less than $30,000	2.1% (*n* = 1)	8.3% (*n* = 1)	2.3% (*n* = 1)	
$30,000–$50,000	2.1% (*n* = 1)	8.3% (*n* = 1)	7.0% (*n* = 3)	8.3% (*n* = 1)
$50,000–$70,000	10.6% (*n* = 5)	16.7% (*n* = 2)	9.3% (*n* = 4)	8.3% (*n* = 1)
More than $70,000	70.2% (*n* = 33)	58.3% (*n* = 7)	72.1% (*n* = 31)	75.0% (*n* = 9)
(Not Reported)	14.9% (*n* = 7)	8.3% (*n* = 1)	9.3% (*n* = 4)	8.3% (*n* = 1)

Note: TD: Typically developing infants; HII: Hypoxic–ischemic injured infants; All percentages rounded to the nearest tenth of a percent and therefore the sum of group percentages within a given category are not always exactly 100%.
